# Attenuated Total Reflection-Fourier Transform Infrared Spectroscopy (ATR-FTIR) Combined with Chemometrics Methods for the Classification of Lingzhi Species

**DOI:** 10.3390/molecules24122210

**Published:** 2019-06-13

**Authors:** Yuan-Yuan Wang, Jie-Qing Li, Hong-Gao Liu, Yuan-Zhong Wang

**Affiliations:** 1 College of Agronomy and Biotechnology, Yunnan Agricultural University, Kunming 650201, China; yuanyuanwang325@163.com (Y.-Y.W.); lijieqing2008@126.com (J.-Q.L.); 2 College of Chinese Medicine, Yunnan University of Chinese Medicine, Kunming 650500, China

**Keywords:** *Ganoderma*, authentication, attenuated total reflection-Fourier transform infrared spectroscopy, chemometrics, random forest, support vector machine, partial least squares discriminant analysis

## Abstract

Due to the existence of Lingzhi adulteration, there is a growing demand for species classification of medicinal mushrooms by various techniques. The objective of this study was to explore a rapid and reliable way to distinguish between different Lingzhi species and compare the influence of data pretreatment methods on the recognition results. To this end, 120 fresh fruiting bodies of Lingzhi were collected, and all of them were analyzed by attenuated total reflection-Fourier transform infrared spectroscopy (ATR-FTIR). Random forest (RF), support vector machine (SVM) and partial least squares discriminant analysis (PLS-DA) classification models were established for raw and pretreated second derivative (SD) spectral matrices to authenticate different Lingzhi species. The results of multivariate statistical analysis indicated that the SD preprocessing method displayed a higher classification ability, which may be attributed to the analysis of powder samples that requires removal of overlapping peaks and baseline shifts. Compared with RF, the results of the SVM and PLS-DA methods were more satisfying, and their accuracies for the test set were both 100%. Among SVM and PLS-DA, the training set and test set accuracy of PLS-DA were both 100%. In conclusion, ATR-FTIR spectroscopy data pretreated by SD combined with PLS-DA is a simple, rapid, non-destructive and relatively inexpensive method to discriminate between mushroom species and provide a good reference to quality assessment.

## 1. Introduction

It is estimated that worldwide there are at least 12,000 species of mushrooms, of which some 2000 species are edible. About 35 species of edible mushroom are commercially grown, while about 200 species of wild edible mushrooms are used for medicinal purposes [1,2]. Medicinal mushrooms have a long history of use in conventional oriental therapies, especially in China, Korea and Japan [3,4,5]. One medicinal mushroom worthy of attention is Lingzhi. Lingzhi is the Chinese name given to the *Ganoderma* family of mushrooms [6,7]. Lingzhi, called “God’s Herb”, are traditional Chinese medicinal fungi, which have been widely used to boost human health and longevity in China and other East Asian countries [8,9]. Modern scientific studies have confirmed that Lingzhi contain fat, dietary fiber, amino acids needed by the human body and many active ingredients such as polysaccharides, ganoderic acid, fatty acids, etc. [10,11,12,13]. The various components display significant therapeutic efficacy, including anti-oxidation, regulation of immunity, inhibition of tumor, and improving the ability of the body to withstand hypoxia activities, useful for the treatment of hypertension, hyperlipidemia, cardiovascular diseases and so forth [14,15]. In addition, various food and health products made from different tissues (mycelia, spores, and fruit body) of Lingzhi with significant properties have acquired enormous commercial value [16,17]. Hence, Lingzhi has a promising value in humans’ daily life.

Different species display different pharmaceutical effects. More than 2000 species of Lingzhi mushrooms are recorded, but only red Lingzhi (*Ganoderma lucidum*) and black Lingzhi (*G. sinensis*) show the most outstanding health-enhancing effects [18], which coincides with the listing of the Pharmacopoeia of the People’s Republic of China (2015 edition) [19]. In other words, the economic value of *G. lucidum* and *G. sinensis* are significant, but their commodity supply chain is complex. Because of the intra-species similarity, adulterated materials are often added fraudulently in the market [20,21]. Traditional recognition methods based on morphological characteristics, mostly depend on professional staff. In some cases, even taxonomists can find correctly identifying plant genera difficult. Authenticity is the foundation for protection of consumer health and sustainable development of Chinese herb medicines. In recent years, the use of herbal products has increased significantly. However, there are also numerous reports of the use of adulterated herbal medicines in many developing countries, which poses a significant threat to public health [22,23]. Because of the lack of an appropriate identification technique, the number of reported cases of fake medicines appears to be increasing [24], so it is significant to correctly distinguish different species Lingzhi to guarantee the quality of Lingzhi products and to prevent their adulteration.

In previous studies, some scholars have used the different triterpenoid contents of Lingzhi mushrooms, dual-mode chromatographic fingerprinting, 63 internal transcribed spacer (ITS) 2 sequences, etc., successfully to distinguish diverse Lingzhi species [25,26,27]. However, these methods require a lot of manpower and complex operations and are time-consuming. Therefore, there is an increasing need for rapid, simple and green methods for the determination of the different Lingzhi species. Spectroscopic methods combined with chemometric methods can solve these problems. Attenuated total reflection-Fourier transform infrared spectroscopy (ATR-FTIR) is widely used in Chinese herbal medicine analysis because it is reliable, rapid, low-cost, nondestructive and allows simultaneous analysis or characterization of various components [28,29]. Chemometric methods, also known as multivariate statistical methods, can highlight the spectral differences between similar samples, model the systematic variance of the data and present it in a simpler way [30,31]. Although chemometric methods combined with ATR-FTIR have been widely applied to classify various food and agricultural products, its use combined with random forest (RF), support vector machine (SVM) and partial least squares discriminant analysis (PLS-DA) chemometric methods in the study of how to classify Lingzhi species is a new challenge.

According to our knowledge, there is no study about the determination of different kinds of Lingzhi species using ATR-FTIR spectroscopy. In this paper, 120 samples collected from Ganodermataceae mushrooms were analyzed by ATR-FTIR. Then RF, SVM and PLS-DA models were applied as classification methods to segregate five different species of Lingzhi samples based on processed spectra data. This study is focused on a comparison of the ability of the three classification models to supply a reliable and rapid method for species classification analysis, and provide quality assessments of Lingzhi.

## 2. Results and Discussion

### 2.1. ATR-FTIR and Pretreatment Spectra Analysis

**Figure 1:**
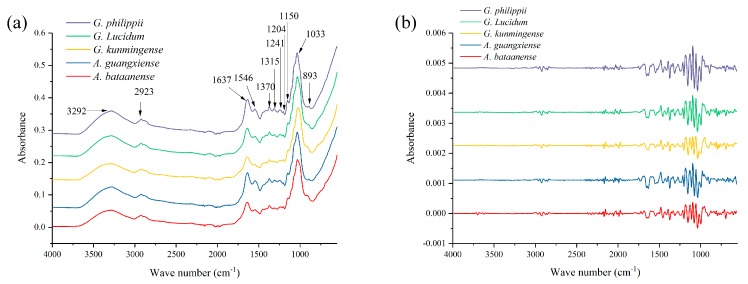
The average spectra of ATR-FTIR of five species of Lingzhi samples. (**a**) Raw ATR-FTIR spectra; (**b**) pretreated SD of the ATR-FTIR spectra.

The ATR-FTIR spectra can provide useful information that identifies the functional groups of the molecules in the samples. Figure S1 displays the raw ATR-FTIR spectra of total Lingzhi samples. Obviously, the spectra of different Lingzhi species samples are similar in shape, although the peak height may differ. From this viewpoint, we can infer that the chemical composition among different species Lingzhi is similar, too. The average spectra of all species are shown in Figure 1a. Several common peaks in these five species spectra were discovered, which allow the construction of profiles of some specific characteristic chemical functional groups. These common peaks were around 3292, 2923, 1637, 1546, 1370, 1315, 1241, 1204, 1150, 1033 and 893 cm^−1^. Based on the literature, we can roughly explain these characteristic absorption bands. The peak at 3292 cm^−1^ represents O-H stretching and N-H stretching, which may come from polysaccharides, triterpenes and sterols. There is a small shoulder absorption around 2923 cm^−1^, which may represent the hydrocarbon chain vibrational mode [32]. The peak at 1637 cm^−1^ is attributed to amide I C=O stretching of the peptide bond. Some weak absorptions appear around 1370, 1315, 1241 and 1204 cm^−1^. The spectral region of 1350–1200 cm^−1^ is attributed to the amide III band [33]. These are due to the absorption of proteins.The peaks in the 1200–950 cm^−1^ range correspond to the characteristic absorption peaks of starch. The ATR-FTIR results preliminarily confirmed that differences exist in the chemical profiles among five different species Lingzhi samples. Figure 1b shows the pretreated SD spectra of the different Lingzhi samples. 

### 2.2. Data Visualization

**Figure 2:**
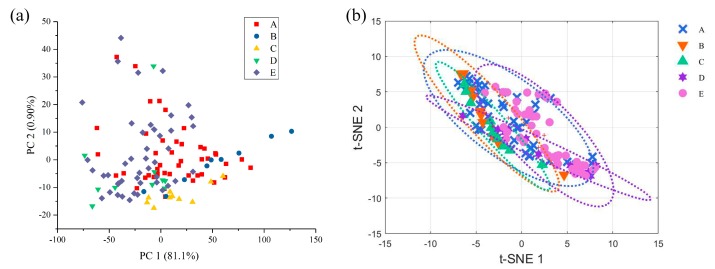
PCA (**a**) and t-SNE (**b**) feature visualization of Lingzhi samples.

The cumulative contribution rate of the first two principal components is shown in Figure S2. The first component represented 81.1% of all sample information. The second component represented 0.90% of the sample information. A PCA score plot based on Fourier transform mid-infrared (FT-MIR) spectra is displayed in Figure 2a. 

T-SNE selected five significant PCs with eigenvalues greater than 1 and provided the visual representation shown in Figure 2b. Visually, the first two PCs and t-SNE can not provide a good enough separation between samples of different species of Lingzhi. The result is possibly due to the fact that raw ATR-FTIR spectra contain not only useful chemical information but also a large amount of noise signals. 

### 2.3. Different Models Established Using ATR-FTIR Spectra

#### 2.3.1. RF Model Established Using ATR-FTIR Spectra

**Figure 3:**
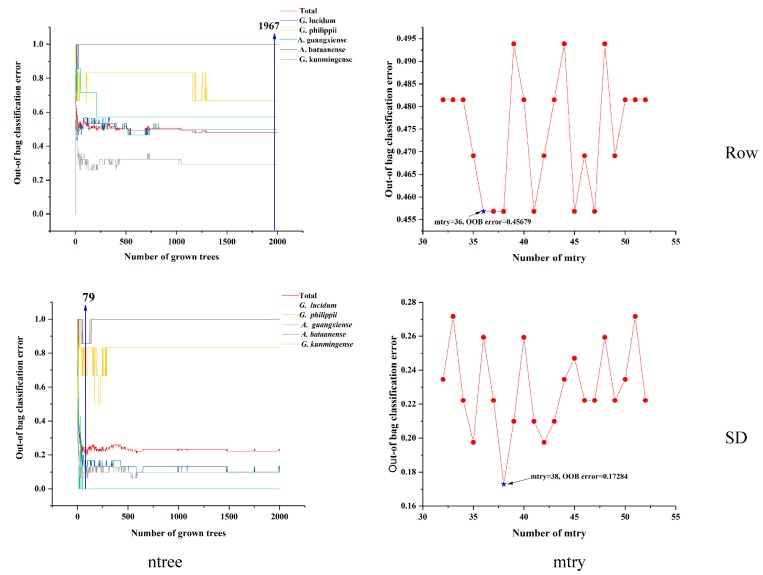
Selection results of tree number and branch node of random forest model using raw and SD spectra.

Independent RF models were built from two dataset sources (raw spectra and spectra pretreated by SD) based on optimal parameters to classify Lingzhi samples according to their different species. The original values of the number of trees (n_tree_) and the number of variables (m_try_) were set as 2000 and square root of the number of all variables, respectively. Therefore, the raw ATR-FTIR spectra m_try_ was set as square root of 1789 and pretreatment SD spectra m_try_ was set as square root of 1775. Figure 3 shows the relationship between the OOB classification error and n_tree_. The optimal number of trees was determined to the one that reached a relatively stable trend at the lowest OOB error, no matter whether total error or each class error. As it could be seen in Figure 3, the OOB error cannot decrease after the number of trees increases, which means the model does not over-fit when the error reaches 1967 and 79. The best n_tree_, m_try_ and the lowest OOB error for raw spectra and SD spectra were 1967, 36 and 0.45679 and 79, 38 and 0.17284, respectively. The two parameter values obviously decreased the error rate from 46.91% to 17.28% after readjustment. 

#### 2.3.2. SVM Model Established Using ATR-FTIR Spectra

**Figure 4:**
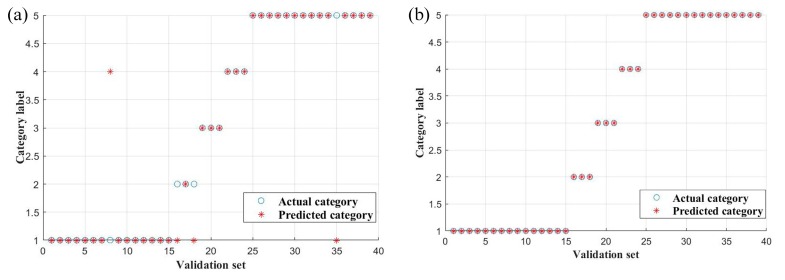
Actual and predicted category results of test samples by SVM. (**a**) Raw, (**b**) SD.

SVM was founded based on the statistical learning theory and structural risk minimization. The SVM technique always gives high prediction rates in a wide range of real applications. The efficiency of SVM and its classification accuracy are highly dependent on the parameter settings [34]. The result for the actual and predicted categories of the test set are shown in Figure 4. In SVM, the kernel parameter (g) and penalty parameter (c), the accuracy of training set and test set were used to evaluate the performance of the classification model. The kernel parameter is closely related to the classification accuracy, and the penalty parameter is the error term. At the same time, the more robust the model is, the lower the penalty parameter is. All parameters of the SVM models are shown in Table 1. The optimal parameters of raw and SD of ATR-FTIR spectra are 5.24288 × 10^5^, 9.5367 × 10^−7^ and 8, 6.9053 × 10^−4^, respectively. Besides, a large value of parameter c implies a high risk of over-fitting for this model. Thus, this result suggested that the SVM model established basing on raw ATR-FTIR spectra data was inadequate to distinguish Lingzhi samples due to the high value of c (risk of overfitting) in the present study. This means spectra pretreated by SD shown less prediction error in the SVM model and the accuracy for the training and test sets are 98.83% and 100%, respectively. Like the results of RF, the classification model established basing on pretreated spectral data is more reliable than the raw spectra one. This demonstrated that spectra pretreated by SD reveal the chemical profile differences among these five species. 

#### 2.3.3. PLS-DA Model Established Using ATR-FTIR Spectra

**Figure 5:**
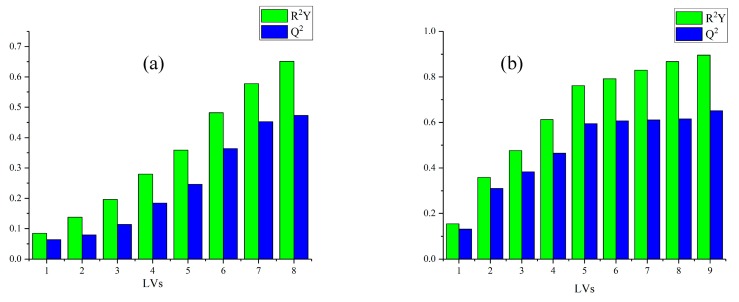
Latent variables (LV_S_), R^2^Y and Q^2^ values of PLS-DA of Raw (**a**) and SD (**b**) spectra for Lingzhi species.

In this paper, ATR-FTIR combined with chemometrics methods were used to classify five species of Lingzhi. In PLS-DA, the parameter R^2^Y represents the cumulative contribution: the higher the value of R^2^Y, the more information is contained in the samples. Furthermore, the parameter Q^2^ is used to evaluate the performance of prediction models, and it indicates a good performance for predicting unknown samples when the value of Q^2^ reaches a maximum. These LVs represent the most information on Lingzhi samples. The first eight LVs of the raw spectra were applied to establish the model. Spectra pretreated by SD used the first nine LVs. These demonstrated that ATR-FTIR spectra contain much information that is irrelevant to the classification Lingzhi samples from different species according to the count of LVs. PLS-DA extracted more effective information from SD spectra than raw spectra. The detailed information of LV_S_, R^2^Y and Q^2^ values of PLS-DA is shown in Figure 5. Moreover, a perfect fit should have low value of root mean square error of estimation (RMSEE), root mean square error of cross validation (RMSECV) and root mean square error of prediction (RMSEP), all of which describe the total error of training and test set. In Table 2, the maximum Q^2^ for the two data sets was 0.651, the R^2^Y was 0.896. In terms of classification accuracy, the training set and test set accuracy of spectra pretreated by SD were both 100%.

The spectra after pretreatment showed a better effect and the classification results of test sets for evaluating the performance of the RF model are shown in Table 3, below. The models based on pretreated spectral data provide more accurate results compared to the model established from raw spectra, which may be attributed to the fact the analysis of powdered samples requires enhancing the resolution and removing the overlapping peaks. This indicated that the SD is a powerful pretreatment method for the classification of Lingzhi samples. According to the classification of each model, parameters of sensitivity, specificity and precision can be calculated. 

The results presented in Table 4 corroborate that the performances of all classification models established using the preprocessed data matrices were better than those of models based on raw spectral data. The sensitivity, specificity, and precision were all equal to 1.00 for all models using SD. Except for the RF model, the correct classification rate was 94.9%, and that of SVM, PLS-DA were 100%. On the other hand, the correct classification of the raw spectra samples was 71.8% for RF, 89.7% for SVM and 89.7% for PLS-DA. As we can see, after pretreating the raw spectra data, the robustness of the models were markedly increased and the risk of overfitting reduced.

## 3. Materials and Methods

### 3.1. Sample Preparation 

A total of 120 fresh fruiting bodies of Lingzhi were collected from Yunnan Province in southwestern China. The collection of five species contained *G. lucidum*, *G. philippii*, *Amauroderma guangxiense*, *A. bataanense*, *G. kunmingense*. Examples. The specific information on these mushroom samples were exhibited in Table 5. All of them were authenticated by Dr. Honggao Liu from the College of Agronomy and Biotechnology, Yunnan Agricultural University, Kunming in China. Initially, the fresh samples were washed, cleaned with soft brush and dried to constant weight in an electrically heated oven (Experimental Instrument Factory, Shanghai, China) at 50 °C for 24 h. Every sample was pulverized in a crusher (FW-100, Tianjin Huaxin Instrument Co., Ltd., Tianjin, China). Then, the powdered samples were passed through an 80-mesh stainless steel sieve. Finally, the sample powders were stored in Zip-Loc bags and stored under dry and room temperature conditions for subsequent analysis.

### 3.2. Spectra Acquisition 

A Fourier transform mid-infrared spectrometer (PerkinElmer, Waltham, MA, USA) equipped with a deuterated triglycine sulfate (DTGS) detector and a golden gate single reflection diamond ATR accessory was used. First, the background spectrum was recorded before the sample spectral information was obtained in order to eliminate any interferences of the external environment and ensure the consistency of the experimental environment. A metal O-ring on diamond crystal was used for sampling. Powders (1.000 ± 0.005 g) were placed on the metal O-ring and 16 scan co-accumulated spectra ranging from 4000 to 550 cm^−1^ with resolution of 4 cm^−1^ were recorded at room temperature. Each sample powder analysis was replicated three times and the average spectra were used for further analysis.

### 3.3. Data Visualization

Two algorithms, principal component analysis (PCA) and t-distributed stochastic neighbor embedding (t-SNE), are used for visualizing high-dimensional data in a two-dimensional representation [35,36]. PCA was carried out using SIMCA-P^+^ 13.0 (Umetrics, Umeå, Sweden) and t-SNE was completed using MATLAB (version R2014a, MathWorks, Natick, MA, USA). Two dimensional PCA was established by two principal components if they contained more than 50% of the data information. T-SNE selects significant PCs with eigenvalues greater than 1, since these PCs could explain more variance than is contained in an original variable.

### 3.4. Data Pretreatment

Many factors such as temperature differences, background disturbance, baseline drift and so on will influence ATR-FTIR spectra. As a result, ATR-FTIR spectra may contain not only useful chemical information, but also physical information which may interfere the spectra. Hence, it is often necessary to preprocess data to minimize the influence of physical effects and enhance the chemical information contribution in model establishment [37,38]. In the literature, we can find many successful examples of quantitative and qualitative analysis of various samples measured with different techniques by derivative spectrometry [39,40,41,42,43,44]. Derivation spectroscopy can eliminate baseline drifts, enhance the resolution and remove the overlap [45,46].

In this paper, we used SD to pretreat the ATR-FTIR spectra. The second derivative greatly enhances the small convexities and concavities of the original (zero order) spectrum, and gives a narrower bandwidth, thus improving the resolution of subtle or overlapping bands [47]. Raw ATR-FTIR spectra data were input into the OMNIC software (Version 8.2, Thermo Fisher Scientific Inc., Waltham, MA, USA) for transforming transmittance into absorbance. Then datasets were established by SIMAC-P^+^ 13.0 (Umetrics) after importing .spc data matrixes to execute the preprocessing procedures. Two-dimensional matrix (m × n) was applied to represent the changing of variable numbers, where m represented the number of samples and n represented their corresponding wavenumbers. The original ATR-FTIR spectra data matrix was consisted of (120 × 1789). After pretreating of SD, data matrix reduced to (120 × 1775) and used for the subsequent chemometric analysis.

### 3.5. Data Analysis

The methods of computational analysis of data can be separated into two kinds: supervised methods (which require a training set of samples to derive a model for dividing the samples into different groups) and unsupervised methods (that do not use previous information from a training set of samples) to classify samples [48]. RF, SVM and PLS-DA are three powerful supervised pattern recognition techniques. Both of them have been successfully used to segregate a variety of samples in combination with spectral techniques. RF has stronger performance in binary classification or regression problems, it was widely applied to studies in the Chinese medicine field [28,49]. The main advantages of SVM are that good results can be obtained even with relatively small datasets, and it can provide a robust classification model and is less affected by the curse of dimensionality and overfitting [50,51]. The main merit of PLS-DA is that the relevant sources of data variability are modeled by latent variables, and the associated PLS scores are then calculated and plotted pairwise, allowing a visualization of group separations [52,53].

Supervised methods require a training set of samples to derive a model for dividing the samples into different groups. Hence, after data preprocessing, samples from each species were separated into a training set (calibration set) containing about two-thirds of the data and a test set (validation set) with one-third via a classical Kennard-Stone algorithm [54]. Finally, a training set containing 81 samples was used to establish the classification model, and 39 samples were used as test set to evaluate the model performance.

RF classifier is a kind of ensemble classifier. It uses a randomly selected subset of training samples and variables to generate multiple decision trees [55]. Over the last two decades, the RF classifier has attracted increasing attention owing to its excellent classification results [56,57]. Establishing an RF model involves the following main steps: after executing the Kennard–Stone (KS) algorithm using MATLAB, we applied the training set to establish the classification trees, and each tree was grown via a pilot sample of raw data. The test set was used to evaluate the performance of the model based on the training set. Original values of the number of trees (n_tree_) and the number of variables (m_try_) were set as 2000 and square root of the number of all variables, respectively. The best numerical values n_tree_ were acquired according to the lowest out-of-bag (OOB) error values. Additionally, the default value of m_try_ was the square root of “number of variables” and the best binary split result of this parameter was used to split the node. Finally, best m_try_ and n_tree_ were used to rerun the above steps to establish a new RF model. Then, the classification model was established and the identification for each sample of test set was obtained. The RF model was established by randomForest (version 3.6.14 ) in R (version 3.4.4) [58].

The SVM algorithm is a linear supervised machine learning method, which can handle non-linear datasets for classification and regression problems. For SVM classification, the underlying concept is to distinguish different classes by an optimal hyperplane in a space. In the practice of non-linear datasets, SVM can’t get an effective classification performance in an original feature space. Under the circumstances, the original data matrix is mapped to a new higher dimensional space using a kernel function, and in this kernel space, samples can be undergone linear identification according to the class labels [59,60]. SVM models were carried out using MATLAB (version R2014a, MathWorks).

PLS-DA is a linear classification method, which combines the properties of partial least squares regression and the discrimination ability of classification techniques [61]. PLS-DA can reduce the effect of multi-collinearity among variables, that is, the lower the collinearity between independent variables, the better the effect of using PLS-DA. In this paper, the signal produced by an ATR-FTIR spectrum is a radiation signal from the surface layer of the sample, the collinearity between radiation signal is low, and PLS-DA can easily recognize system information and noise, which can lead to a better classification effect [62]. A fundamental step to build a PLS-DA model is the determination of the number of latent variables (LVs). This choice is commonly performed by using cross-validation of the training samples where some samples are separated into a test set and the models are built with the others. The prediction errors are calculated for the samples that were separated using different numbers of latent variables. The process is repeated until all samples are predicted [63]. PLS-DA was carried out using SIMCA-P^+^ 10.0 (Umetrics).

Performance of discrimination model was estimated by means of sensitivity, specificity and precision. The parameters were dependent on four values: true positive (TP), false negative (FN), true negative (TN), and false positive (FP). Positive means itself, and negative indications are classified into other class. TP was the positive samples correctly classified into positive class. FN was the positive samples that were classified negative class, TN was the negative samples that were correctly classified negative class, FP was the number of negative samples that were classified as positive class [64]:

$$\mathrm{Sensitivity}\;=\;\frac{\mathrm{TP}}{\mathrm{TP}+\mathrm{FN}}$$

$$\mathrm{Specificity}\;=\;\frac{\mathrm{TN}}{\mathrm{TN}+\mathrm{FP}}$$

$$\mathrm{Precision}\;=\;\frac{\mathrm{TP}}{\mathrm{TP}+\mathrm{FP}}$$

Sensitivity shows model ability to correctly distinguish samples belonging to that class. Specificity reflects the model ability to exclude samples belonging to all other classes. A further evaluation parameter is the precision of the model which was defined as the percentage of the number of samples correctly classified in the total of samples. These indexes can illustrate as the probability that a positive classification will be correct classified [65,66].

## 4. Conclusions

Correct discrimination of Lingzhi species is very important to ensure the clinical medication safety and efficiency, as well as the species identification. This study revealed the powerful ability of the second derivative spectra to improve the accuracy of classification models. Supervised analysis approaches including RF, SVM and PLS-DA were applied for investigating the classification performance based on the ATR-FTIR spectra of 120 Lingzhi samples belonging to five species. Subsequently, the classification performance of three supervised methods were compared. The chemometric PLS-DA method showed the most satisfying results. Hence, this study demonstrated that ATR-FTIR coupled with PLS-DA was an effective and accurate method to discriminate Lingzhi mushrooms with the help of spectra pretreated by SD. 

## Figures and Tables

**Table 1:** Results of SVM models for recognizing different species of Lingzhi basing on different data matrices.

Data Matrices	Best c	Best g	Accuracy of Training Set (%)	Accuracy of Test Set (%)
Raw	5.24288 × 10^5^	9.5367 × 10^−7^	82.72	89.74
SD	8	6.9053 × 10^−^^4^	93.83	100

**Table 2:** Results of PLS-DA models of different species basing on different data matrices.

Data Matrices	Rmsee	Rmsecv	Rmsep	Q^2^	R^2^Y	Accuracy of Training Set (%)	Accuracy of Test Set (%)
Raw	0.217228	0.25516	0.210544	0.474	0.651	92.59	89.74
SD	0.12055	0.22003	0.120649	0.651	0.896	100	100

**Table 3:** Summary of the classification of Lingzhi by different models and spectra pretreatment using ATR-FTIR spectra.

Methods	Predicted	Raw					SD				
		A	B	C	D	E	A	B	C	D	E
RF	A	11	0	0	0	4	15	0	0	0	0
	B	1	2	0	0	0	0	3	0	0	0
	C	2	0	1	0	0	0	0	3	0	0
	D	0	0	0	0	3	2	0	0	1	0
	E	1	0	0	0	14	0	0	0	0	15
SVM	A	14	0	0	1	0	15	0	0	0	0
	B	2	1	0	0	0	0	3	0	0	0
	C	0	0	3	0	0	0	0	3	0	0
	D	0	0	0	3	0	0	0	0	3	0
	E	1	0	0	0	14	0	0	0	0	15
PLS-DA	A	15	0	0	0	0	15	0	0	0	0
	B	1	2	0	0	0	0	3	0	0	0
	C	0	0	3	0	0	0	0	3	0	0
	D	0	0	0	1	2	0	0	0	3	0
	E	1	0	0	0	14	0	0	0	0	15

**Table 4:** Parameters of merit for the classification of Lingzhi using ATR-FTIR spectrum after applying different chemometric methods and spectra pretreatment.

Methods	Parameter	Raw					SD				
		A	B	C	D	E	A	B	C	D	E
RF	Sensitivity	0.733	0.667	0.333	0.000	0.933	1.000	1.000	1.000	0.333	1.000
	Specificity	0.833	1.000	1.000	1.000	0.708	0.917	1.000	1.000	1.000	1.000
	Precision	0.733	1.000	1.000	0.000	0.667	0.882	1.000	1.000	1.000	1.000
SVM	Sensitivity	0.933	0.333	1.000	1.000	0.933	1.000	1.000	1.000	1.000	1.000
	Specificity	0.875	1.000	1.000	0.972	1.000	1.000	1.000	1.000	1.000	1.000
	Precision	0.824	1.000	1.000	0.75	1.000	1.000	1.000	1.000	1.000	1.000
PLS-DA	Sensitivity	1.000	0.667	1.000	0.333	0.933	1.000	1.000	1.000	1.000	1.000
	Specificity	0.917	1.000	1.000	1.000	0.917	1.000	1.000	1.000	1.000	1.000
	Precision	0.882	1.000	1.000	1.000	0.875	1.000	1.000	1.000	1.000	1.000

**Table 5:** Information of the mushroom samples.

Code	Quantity	NO.	Latin Name
A	45	1–45	*G. lucidum*
B	9	46–54	*G. philippii*
C	10	55–64	*A. guangxiense*
D	10	65–74	*A. bataanense*
E	46	75–120	*G. kunmingense*

## Supplementary Materials

The following are available online; Figures S1 and S2.

## References

[1] Aida F.M.N.A., Shuhaimi M., Yazid M., Maaruf A.G. (2009). Mushroom as a potential source of prebiotics: A review. Trends Food Sci. Technol..

[2] Rathore H., Prasad S., Sharma S. (2017). Mushroom nutraceuticals for improved nutrition and better human health: A review. PharmaNutrition.

[3] Wang X., Zhang J., Wu L., Zhao Y., Li T., Li J., Wang Y., Liu H. (2014). A mini-review of chemical composition and nutritional value of edible wild-grown mushroom from China. Food Chem..

[4] Kim M., Seguin P., Ahn J., Kim J., Chun S., Kim E., Seo S., Kang E., Kim S., Park Y. (2008). Phenolic Compound Concentration and Antioxidant Activities of Edible and Medicinal Mushrooms from Korea. J. Agric. Food Chem..

[5] Zaidman B., Yassin M., Mahajna J., Wasser S.P. (2005). Medicinal mushroom modulators of molecular targets as cancer therapeutics. Appl. Microbiol. Biol..

[6] Cao Y., Wu S., Dai Y. (2012). Species clarification of the prize medicinal Ganoderma mushroom “Lingzhi”. Fungal Divers..

[7] Yang Z.L., Feng B. (2013). What is the Chinese “Lingzhi”?—A taxonomic mini-review. Mycology.

[8] Bao X.F., Wang X.S., Dong Q., Fang J.N., Li X.Y. (2002). Structural features of immunologically active polysaccharides from Ganoderma lucidum. Phytochemistry.

[9] Zheng Y., Limin H., Liming Z., Caicai K., Tianjiao M., Yan C. (2019). Comparison of Main Chemical Constituents in Ganoderma lucidum Collected from Three Producing Districts. Food Sci..

[10] Jong S.C., Birmingham J.M. (1992). Medicinal benefits of the mushroom Ganoderma. Adv. Appl. Microbiol..

[11] Jin M.L., Zhang H., Wang J.J., Shao D., Yang H., Huang Q., Shi J., Xu C., Zhao K. (2019). Response of intestinal metabolome to polysaccharides from mycelia of Ganoderma lucidum. Int. J. Biol. Macromol..

[12] Kubota T., Asaka Y., Miura I., Mori H. (1982). Structures of Ganoderic Acid A and B, Two New Lanostane Type Bitter Triterpenes from Ganoderma lucidum (FR.). Helv. Chim. Acta.

[13] Boh B., Berovic M., Zhang J., Zhi-Bin L. (2007). Ganoderma lucidum and its pharmaceutically active compounds. Biotechnol. Annu. Rev..

[14] Holmes D. (2015). Medicinal mushroom reduces obesity by modulating microbiota. Nat. Rev. Endocrinol..

[15] Cör D., Knez Ž., Knez Hrnčič M. (2018). Antitumour, Antimicrobial, Antioxidant and Antiacetylcholinesterase Effect of Ganoderma Lucidum Terpenoids and Polysaccharides: A Review. Molecules.

[16] Wachtel-Galor S., Yuen J., Buswell J.A., Benzie I.F.F. (2011). Ganoderma lucidum (Lingzhi or Reishi). Herb. Med..

[17] Babu P.D., Subhasree R.S. (2008). The Sacred Mushroom “Reishi”—A Review. J. Bot..

[18] Richter C., Wittstein K., Kirk P.M., Stadler M. (2015). An assessment of the taxonomy and chemotaxonomy of Ganoderma. Fungal Divers..

[19] Chinese Pharmacopoeia Commission (2015). Chinese Pharmacopoeia.

[20] Shao P., Wang J., Zhang T., Sun P. (2015). Determination of starch adulteration in Ganoderma lucidum polysaccharide by near infrared reflectance spectroscopy with partial least squares algorithm. Curr. Top. Nutraceut. Res..

[21] Fu H., Yin Q., Xu L., Wang W., Chen F., Yang T. (2017). A comprehensive quality evaluation method by FT-NIR. Spectrochim. Acta A.

[22] Han J., Pang X., Liao B., Yao H., Song J., Chen S. (2016). An authenticity survey of herbal medicines from markets in China using DNA barcoding. Sci. Rep..

[23] Gautam C.S., Utreja A., Singal G.L. (2009). Spurious and counterfeit drugs: A growing industry in the developing world. Postgrad. Med. J..

[24] Marini R.D., Rozet E., Montes M.L., Rohrbasser C., Roht S., Rheme D., Bonnabry P., Schappler J., Veuthey J.L., Hubert P. (2010). Reliable low-cost capillary electrophoresis device for drug quality control and counterfeit medicines. J. Pharm. Biomed. Anal..

[25] Su C.H., Yang Y.Z., Ho H.O., Hu C.H., Sheu M.T. (2001). High-performance liquid chromatographic analysis for the characterization of triterpenoids from Ganoderma. J. Chromatogr. Sci..

[26] Chen Y., Bicker W., Wu J., Xie M.Y., Lindner W. (2010). Ganoderma species discrimination by dual-mode chromatographic fingerprinting: A study on stationary phase effects in hydrophilic interaction chromatography and reduction of sample misclassification rate by additional use of reversed-phase chromatography. J. Chromatogr. A.

[27] Liao B., Chen X., Han J., Dan Y., Wang L., Jiao W., Song J., Chen S. (2015). Identification of commercial Ganoderma (Lingzhi) species by ITS2 sequences. Chin. Med..

[28] Pei Y., Wu L., Zhang Q., Wang Y. (2019). Geographical traceability of cultivatedParis polyphylla var.yunnanensis using ATR-FTMIR spectroscopy with three mathematical algorithms. Anal. Methods.

[29] Wang Y., Huang H., Zuo Z., Wang Y. (2018). Comprehensive quality assessment of Dendrubium officinale using ATR-FTIR spectroscopy combined with random forest and support vector machine regression. Spectrochim. Acta A.

[30] de Oliveira Magalhães L., Arantes L.C., Willian J., Braga B. (2019). Identification of NBOMe and NBOH in blotter papers using a handheld NIR spectrometer and chemometric methods. Microchem. J..

[31] Cebi N., Dogan C.E., Mese A.E., Ozdemir D., Arıcı M., Sagdic O. (2019). A rapid ATR-FTIR spectroscopic method for classification of gelatin gummy candies in relation to the gelatin source. Food Chem..

[32] Yancheva D., Tapanov S., Velcheva E., Stamboliyska B., Glavcheva Z., Stoyanov S., Haralampiev N., Fischer D., Lederer A. (2017). Characterization of Zahari Zograph’s nave wall paintings in the church “The nativity of the virgin” of Rila Monastery (Bulgaria) by vibrational spectroscopy and SEM–EDX analysis. Sci. Tech. Arch. Res..

[33] Cai S., Singh B.R. (1999). Identification of β-turn and random coil amide III infrared bands for secondary structure estimation of proteins. Biophys. Chem..

[34] Faris H., Hassonah M.A., Al-Zoubi A.M., Mirjalili S., Aljarah I. (2018). A multi-verse optimizer approach for feature selection and optimizing SVM parameters based on a robust system architecture. Neural Comp. Appl..

[35] van der Maaten L., Hinton G. (2008). Visualizing data using t-SNE. Mach. Learn. Res..

[36] Gorban A.N., Kégl B., Wunsch D.C., Zinovyev A.Y. (2008). Principal Manifolds for Data Visualization and Dimension Reduction.

[37] Shen F., Yang D., Ying Y., Li B., Zheng Y., Jiang T. (2012). Discrimination Between Shaoxing Wines and Other Chinese Rice Wines by Near-Infrared Spectroscopy and Chemometrics. Food Bioprocess Technol..

[38] Li Y., Zhang J., Li T., Liu H., Li J., Wang Y. (2017). Geographical traceability of wild Boletus edulis based on data fusion of FT-MIR and ICP-AES coupled with data mining methods (SVM). Spectrochim. Acta A.

[39] Zhang J., Yan Y. (2005). Probing conformational changes of proteins by quantitative second-derivative infrared spectroscopy. Anal. Biochem..

[40] Genkawa T., Ahamed T., Noguchi R., Takigawa T., Ozaki Y. (2016). Simple and rapid determination of free fatty acids in brown rice by FTIR spectroscopy in conjunction with a second-derivative treatment. Food Chem..

[41] Cielecka-Piontek J. (2013). Derivative Spectrophotometry for the Determination of Faropenem in the Presence of Degradation Products: An Application for Kinetic Studies. Appl. Spectrosc..

[42] Mathian M., Hebert B., Baron F., Petit S., Lescuyer J.L., Furic R., Beaufort D. (2018). Identifying the phyllosilicate minerals of hypogene ore deposits in lateritic saprolites using the near-IR spectroscopy second derivative methodology. J. Geochem. Explor..

[43] Barreca S., Mazzola A., Orecchio S., Tuzzolino N. (2014). Polychlorinated Biphenyls in Sediments from Sicilian Coastal Area (Scoglitti) using Automated Soxhlet, GC-MS, and Principal Component Analysis. Polycycl. Aromat. Comp..

[44] Amorello D., Orecchio S., Pace A., Barreca S. (2016). Discrimination of almonds (Prunus dulcis) geographical origin by minerals and fatty acids profiling. Nat. Prod. Res..

[45] Li D., Jin Z., Zhou Q., Chen J., Lei Y., Sun S. (2010). Discrimination of five species of Fritillaria and its extracts by FT-IR and 2D-IR. J. Mol. Struct..

[46] Czarnecki M.A. (2015). Resolution Enhancement in Second-Derivative Spectra. Appl. Spectrosc..

[47] Kosmas C.S., Curi N., Bryant R.B., Franzmeier D.P. (1984). Characterization of Iron Oxide Minerals by Second-Derivative Visible Spectroscopy1. Soil Sci. Soc. Am. J..

[48] Berrueta L.A., Alonso-Salces R.M., Héberger K. (2007). Supervised pattern recognition in food analysis. J Chromatogr. A.

[49] Li Y., Zhang J., Wang Y. (2018). FT-MIR and NIR spectral data fusion: A synergetic strategy for the geographical traceability of Panax notoginseng. Anal. Bioanal. Chem..

[50] Villa J.E.L., Quiñones N.R., Fantinatti-Garboggini F., Poppi R.J. (2019). Fast discrimination of bacteria using a filter paper–based SERS platform and PLS-DA with uncertainty estimation. Anal. Bioanal. Chem..

[51] Devos O., Downey G., Duponchel L. (2014). Simultaneous data pre-processing and SVM classification model selection based on a parallel genetic algorithm applied to spectroscopic data of olive oils. Food Chem..

[52] Paiva J.S., Cardoso J., Pereira T.A. (2018). Supervised Learning Methods for Pathological Arterial Pulse Wave. Int J. Med. Inform..

[53] Barker M., Rayens W. (2003). Partial least squares for discrimination. J. Chemometr..

[54] Rajer-Kanduč K., Zupan J., Majcen N. (2003). Separation of data on the training and test set for modelling: A case study for modelling of five colour properties of a white pigment. Chemometr. Intell. Lab..

[55] Belgiu M., Drăguţ L. (2016). Random forest in remote sensing: A review of applications and future directions. ISPRS J. Photogramm..

[56] Breiman L. (2001). Random forests. Mach. Learn..

[57] Genuer R., Poggi J., Tuleau-Malot C., Villa-Vialaneix N. (2017). Random Forests for Big Data. Big Data Res..

[58] Liaw A., Wiener M. (2002). Classification and Regression by RandomForest. R News.

[59] Li Y., Wang Y. (2018). Synergistic strategy for the geographical traceability of wild Boletus tomentipes by means of data fusion analysis. Microchem. J..

[60] Kotsiantis S.B. (2007). Supervised machine learning: A review of classification techniques. Emerg. Artif. Intell. Appl. Comp. Eng..

[61] Ballabio D., Consonnia V. (2013). Classification tools in chemistry. Part 1: Linear models. PLS-DA. Anal. Methods.

[62] Górski Ł., Sordoń W., Ciepiela F., Kubiak W.W., Jakubowska M. (2016). Voltammetric classification of ciders with PLS-DA. Talanta.

[63] de Almeida M.R., Correa D.N., Rocha W.F.C., Scafi F.J.O., Poppi R.J. (2013). Discrimination between authentic and counterfeit banknotes using Raman spectroscopy and PLS-DA with uncertainty estimation. Microchem. J..

[64] Luna A.S., Da Silva A.P., Da Silva C.S., Lima I.C.A., de Gois J.S. (2019). Chemometric methods for classification of clonal varieties of green coffee using Raman spectroscopy and direct sample analysis. J Food Compos. Anal..

[65] Oliveri P., Downey G. (2012). Multivariate class modeling for the verification of food-authenticity claims. TrAC Trends Anal. Chem..

[66] Brereton R.G. (2009). Chemometrics for Pattern Recognition.

