# Supplementary material for: Attenuated Total Reflection-Fourier Transform Infrared Spectroscopy (ATR-FTIR) Combined with Chemometrics Methods for the Classification of Lingzhi Species
Source: Molecules. 2019 Jun 13;24(12):2210. doi: 10.3390/molecules24122210 (PMC6631843; doi:10.3390/molecules24122210)
Supplement: Supplementary file 1 [file molecules-24-02210-s001.pdf]

Attenuated total reflection-Fourier transform infrared spectroscopy (ATR-FTIR) combined with chemometrics methods for the classification of Lingzhi species

Yuan-Yuan Wang[1,2], Jie-Qing Li[1], Hong-Gao Liu[1,*] and Yuan-Zhong Wang[2,*]

1 College of Agronomy and Biotechnology, Yunnan Agricultural University, Kunming 650201, China;yuanyuanwang325@163.com

2 Institute of Agro-Products Processing Science and Technology, Yunnan Academy of Agricultural Sciences, Kunming 650221, China

[*] Correspondence: honggaoliu@126.com (H.-G.L.); Tel:+86-871-65221696; boletus@126.com (Y.-Z.W.); Tel:86-871-65033575

Figure captions

Figure S1 Row ATR-FTIR spectra of 120 Lingzhi samples.

Figure S2. Principle (PCs), R^2X, Q^2 value of two dimension PCA.

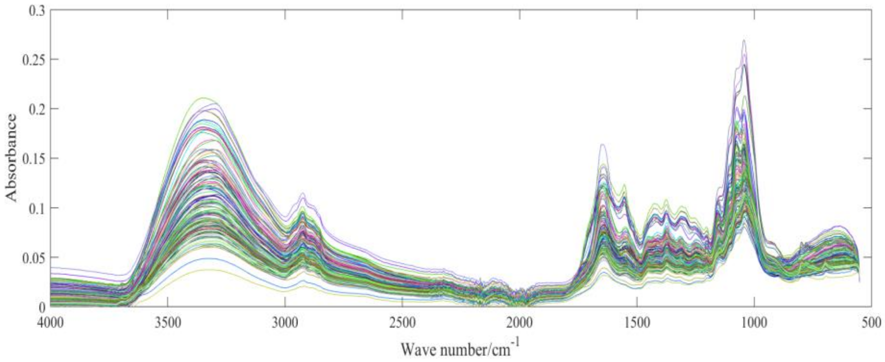
0.3
0.25
0.2
0.15
0.1
0.05
0
Absorbance
4000
3500
3000
2500
2000
1500
1000
500
Wave number/cm-1

Figure S1

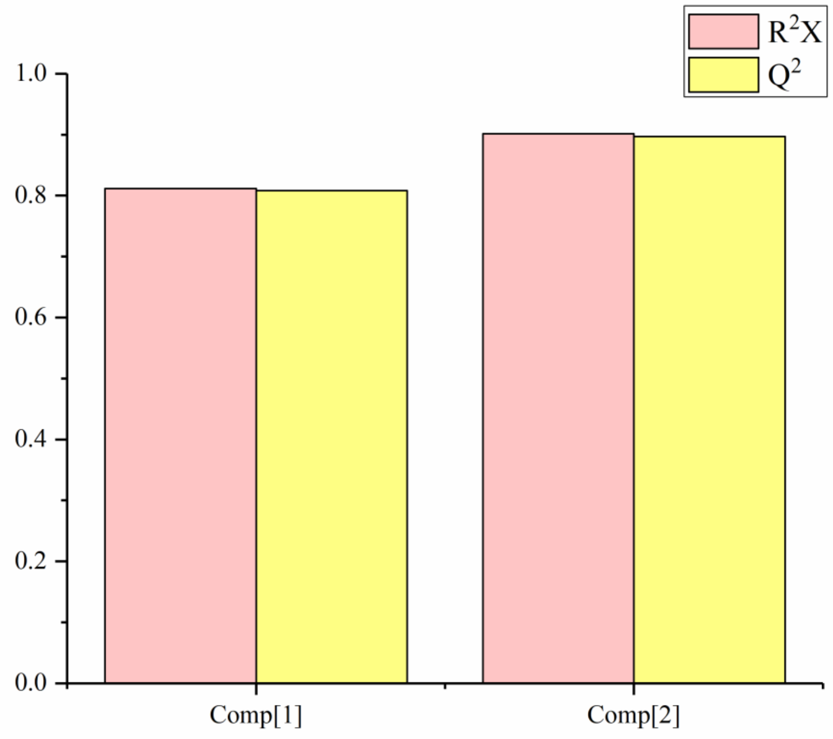

Figure S2
